# Leishmanicidal Activity of Betulin Derivatives in *Leishmania amazonensis;* Effect on Plasma and Mitochondrial Membrane Potential, and Macrophage Nitric Oxide and Superoxide Production

**DOI:** 10.3390/microorganisms9020320

**Published:** 2021-02-04

**Authors:** Wilmer Alcazar, Sami Alakurtti, Maritza Padrón-Nieves, Maija Liisa Tuononen, Noris Rodríguez, Jari Yli-Kauhaluoma, Alicia Ponte-Sucre

**Affiliations:** 1 Laboratory of Molecular Physiology, Institute of Experimental Medicine, School of Medicine Luis Razetti, Faculty of Medicine, Universidad Central de Venezuela, P.O. Box 50587, Caracas 1050, Venezuela; alcazarwilmer@gmail.com (W.A.); mpadron43@gmail.com (M.P.-N.); 2 Drug Research Program, Division of Pharmaceutical Chemistry and Technology, Faculty of Pharmacy, University of Helsinki, Viikinkaari 5 E, P.O. Box 56, FI-00014 Helsinki, Finland; sami.alakurtti@neste.com (S.A.); pkbpka@mbnet.fi (M.L.T.); 3 VTT Technical Research Centre of Finland, Biologinkuja 7, P.O. Box 1000, FI-02044 Espoo, Finland; 4 Laboratory of Genetic Engineering, Institute of Biomedicine, Universidad Central de Venezuela, P.O. Box 4043, Caracas 1010A, Venezuela; nmrodric@gmail.com

**Keywords:** *Leishmania*, betulin derivatives, plasma membrane potential, mitochondrial membrane potential, therapeutic failure, drug resistance

## Abstract

Herein, we evaluated in vitro the anti-leishmanial activity of betulin derivatives in Venezuelan isolates of *Leishmania amazonensis*, isolated from patients with therapeutic failure. Methods: We analyzed promastigote in vitro susceptibility as well as the cytotoxicity and selectivity of the evaluated compounds. Additionally, the activity of selected compounds was determined in intracellular amastigotes. Finally, to gain hints on their potential mechanism of action, the effect of the most promising compounds on plasma and mitochondrial membrane potential, and nitric oxide and superoxide production by infected macrophages was determined. Results: From the tested 28 compounds, those numbered **18** and **22** were chosen for additional studies. Both **18** and **22** were active (GI_50_ ≤ 2 µM, cytotoxic CC_50_ > 45 µM, SI > 20) for the reference strain LTB0016 and for patient isolates. The results suggest that **18** significantly depolarized the plasma membrane potential (*p* < 0.05) and the mitochondrial membrane potential (*p* < 0.05) when compared to untreated cells. Although neither **18** nor **22** induced nitric oxide production in infected macrophages, **18** induced superoxide production in infected macrophages. Conclusion: Our results suggest that due to their efficacy and selectivity against intracellular parasites and the potential mechanisms underlying their leishmanicidal effect, the compounds **18** and **22** could be used as tools for designing new chemotherapies against leishmaniasis.

## 1. Introduction

Leishmaniasis is endemic in 98 countries, in which 14 million people remain infected and more than 350 million are at risk [1,2]. Approximately 1.3 million people contract the disease annually; 300,000 cases correspond to visceral leishmaniasis (VL) and 1,000,000 to tegumentary leishmaniasis (TL). The estimated number of deaths from VL ranges between 20,000 and 50,000 each year [3]. However, these numbers reflect the tip of the iceberg, since only 40 countries where the disease is endemic regularly report their health statistics to the World Health Organization (WHO) [4].

In Venezuela, VL has a low incidence (0.03 to 0.24 per 100,000 inhabitants) [5], although a notable under-registration of cases remains [6]. The main focus of VL is located at the Nueva Esparta State [5], a geographical area in which the whole life cycle of *Leishmania (L.) infantum* has been described, including the dog as the main reservoir, as well as *Lutzomyia longipalpis* as the insect vector [7]. TL is officially recognized as a public health problem [5,8]. A total of 66,550 cases in its various clinical forms, with an annual incidence rate that varied between 5.3 per 100,000 inhabitants (2014) and 12 per 100,000 inhabitants (1992), have been reported during the period 1990–2018. TL represents 99% of the cases of leishmaniasis reported each year, of which 98% correspond to localized cutaneous leishmaniasis (LCL), 1% mucocutaneous leishmaniasis (MCL), and 0.2% diffuse cutaneous leishmaniasis (DCL) [9].

After malaria, leishmaniasis is the protozoan parasitic disease with the highest prevalence worldwide (14 million people infected). It is the second in number of disability-adjusted life years or healthy years of life lost (1.7 million), the second with the highest mortality rate (up to 50,000 deaths per year), and the third cause of morbidity due to communicable diseases (1,300,000 new cases per year), after malaria and tuberculosis [1,2,3,4].

There are no pharmacological treatments sufficiently effective, safe, and affordable to combat diseases caused by kinetoplastid parasites, including *Leishmania* [2,10]. Progress in the biology, epidemiology, and immunology of leishmaniasis and the characterization of the parasite genome and proteome form the framework to design leishmaniasis chemotherapy of the future. Approaches to the development of chemotherapeutic strategies against the disease include optimization of the available therapy, use of active compounds against other diseases, pharmacological target-oriented design, and evaluation of natural products and their derivatives.

Prospects for developing an effective leishmaniasis vaccine are not immediate and disease control is based primarily on chemotherapeutic treatment, although the arsenal of available drugs is reduced and with significant associated toxicity; vector control is problematic generating environmental risk; comorbidity with HIV is increasing; and finally, almost all endemic regions exhibit a growing incidence of therapeutic failure [11,12,13,14]. Consequently, the design of more effective and safer therapeutic alternatives has become an extraordinary and urgent need [15,16,17]. Meanwhile, there is a delay in the development of new drugs, mainly due to the lack of economic stimuli that this disease represents for the pharmaceutical companies. For example, in the 2000s, the global investment in new antiparasitic drugs was only about 0.1% of the global asset in research [18]. Currently, with the exception of miltefosine (MLF), the same anti-leishmanial drugs of the early last century are still used.

Experimental development of new anti-leishmanial agents either starts from a validated target or they are searched by phenotypic screening [19]. The first approach has proved to be unsuccessful, because a powerful inhibitory agent found against an enzyme or another particular target usually fails to have activity in the entire microorganism [20]. The second alternative involves all possible therapeutic targets and simultaneously allows evaluation of compound activity in the cellular environment, in a much more real condition. Thus, phenotypic screening that includes in vitro susceptibility tests and the macrophage-amastigote model stands out as the cornerstone of studies prior to the development of new chemotherapeutics against this disease [21]. For example, trifluoromethyl-substituted quinolones and their analogues, as well as metronidazole derivatives have emerged as an interesting platform to design agents against *L. (L.) mexicana* and *L. (V.) braziliensis* [22,23].

According to the WHO, plants are the largest source of medicines for humans [24]. In fact, natural products and their derivatives are the source of 30% of the world pharmaceutical market [25]. In this scenario, extracts or compounds of plant origin, including terpenes, phenolics, and alkaloids, can be used as a valuable starting point for the search for new anti-leishmanial agents [18]. Among terpenoids, betulin, a pentacyclic triterpene, isolated from the bark of trees of the genus *Betula* (birches), stands out [26]. The leishmanicidal potential of some betulin derivatives has already been documented in the literature [27,28,29,30]. However, for this type of compounds, their effectiveness in New World species, or in parasites isolated from patients with therapeutic failure, is not clear, nor is their mechanism of action well known.

Herein, we evaluated in vitro the anti-leishmanial activity of betulin derivatives in Venezuelan isolates of *Leishmania amazonensis* [*L. (L.) amazonensis*], isolated from patients with therapeutic failure. Our results suggest that, due to their efficacy and selectivity against intracellular parasites and the mechanisms underlying their leishmanicidal effect, some of these compounds could be used as tools for designing new chemotherapies against leishmaniasis.

## 2. Materials and Methods

### 2.1. Parasites

Three *L. (L.) amazonensis* isolates were used: a reference species certified by the WHO (MHOM/BR/77/LTB0016, LTB0016) donated by Dr. Lionel Schnur, Hebrew University-Hadassah Medical School, Jerusalem, Israel, and two isolates (MHOM/VE/1998/MR, VE98MR; and MHOM/VE/2000/MM, VE2000MM) obtained from patient lesions identified as such at the Dermatology Department, Institute of Biomedicine (SAIB-MPPS-UCV), Caracas, Venezuela. The patients were diagnosed DCL and suffered therapeutic failure against meglumine antimoniate (Glucantime^®^, Sanofi-Aventis (Suzano, SP, Brazil). Species identification was performed by PCR and molecular hybridization [31]. This project (CDCH UCV PI-09-8717-2013) was approved by the Ethics Committee from the Institute of Biomedicine (SAIB-MPPS-UCV), Caracas, Venezuela. Isolates were kept in liquid nitrogen until use, when parasites were thawed and cultured in blood agar-glucose enriched with human urine [32,33].

*Leishmania* promastigotes were grown at 26 °C in semisolid blood agar supplemented with glucose-NaCl medium (glucose 1.5%, NaCl 0.85%, *w*/*v*). Promastigotes at late log growth phase were collected by centrifugation at 125× *g* for 10 min at room temperature (RT). The medium was decanted and the cells were suspended in the appropriate medium up to the desired cell density.

### 2.2. Murine Macrophages

J774.1 macrophages [34], donated by Dr. Concepción Hernández, Institute of Experimental Biology-Universidad Central de Venezuela, were used. Culture monolayers were established in complete Roswell Park Memorial Institute medium (RPMIc; 25 mM HEPES, 2 mM L-glutamine, 5 mg L^−1^ phenol red, supplemented with 10% fetal calf serum, 2 g L^−1^ NaHCO_3_, pH 7.2, 0.02 mM 2-mercaptoethanol, and 100 U mL^−1^ penicillin plus 100 µg mL^−1^ streptomycin), at 37 °C and 5% CO_2_. Culture medium was replaced weekly. For the experimental procedures, cells were detached from the flasks by mechanical procedures, washed twice with phosphate-buffered saline (NaCl 136 mM, KCl 2.6 mM, Na_2_HPO_4_⋅2H_2_O 10 mM, KH_2_PO_4_ 1.7 mM; PBS, pH 7.4), and suspended at the desired density in RPMIc.

### 2.3. Compounds

Twenty-eight semisynthetic betulin derivatives (20 structurally simple and 8 heterocyclic derivatives, compounds **1**–**20** and **21**–**28**, respectively) were tested. The compounds, stable at RT, were dissolved in DMSO at a concentration of 10 mM and stored at −20 °C until use. Their synthesis and properties have been described by Alcazar et al. and references therein [28]. A summary of their chemical formulae, structures, and molecular weights is included in Supplementary Materials I.

### 2.4. Promastigote In Vitro Susceptibility

The fluorometric method of Alamar Blue^®^ (resazurin) was used [35]. Promastigotes grown in biphasic agar-blood medium, at the exponential phase of growth, were seeded in flat-bottom 96-well culture plates, at a cell density of 1 × 10^6^ parasites mL^−1^ (200,000 parasites/well), in 180 µL of RPMIc without phenol red. Subsequently, either a sole concentration (50 µM), or increasing concentrations of compounds or drugs were applied, according to each treatment group, in volumes of 2 µL. Plates were kept at 26 °C, in a humid chamber, for 72 h. Resazurin was then added (20 µL of 10% *v*/*v*), to reach a final concentration of 250 µM and plates were incubated for an additional 4 h. Live parasites are metabolically active and capable of reducing non-fluorescent resazurin (blue) to fluorescent resorufin (red). Fluorescence intensity is directly proportional to the number of viable cells. The fluorometric reading was carried out using the appropriate wavelengths (ex: 540 nm/em: 580 nm) in a spectrofluorometer Victor^2^ Wallac (Perkin Elmer, Turku, Finland). At least two independent experiments were performed in duplicate. The effect of trivalent antimonial [SbIII, potassium antimonyl tartrate trihydrate, Sigma-Aldrich, (St. Louis, MO, USA)] was included as positive control of the assay. Based on growth inhibition caused by the tested compounds, the percentage of growth inhibition compared to untreated cells was used to calculate the GI_50_ as the concentration that causes 50% inhibition of cell proliferation [36].

### 2.5. Cytotoxicity and Selectivity

Cytotoxicity of “active compounds” was evaluated by the fluorometric method of Alamar Blue^®^ [37]. In flat-bottom 96-well culture plates, J774.1 macrophages were seeded at a cell density of 5 × 10^4^ mL^−1^ (10,000 macrophages/well) in 200 µL of RPMIc. After 24 h at 37 °C, 5% CO_2_, medium was replaced by 180 µL of RPMIc, without phenol red, and increasing concentrations of test compounds in 2-µL volumes. Plates were kept at 37 °C, 5% CO_2_ for 72 h; then resazurin was added (20 µL, 10% *v*/*v*) to a final concentration of 250 µM and plates were incubated for additional 4 h. Fluorometric readings were carried out using the appropriate wavelengths (ex: 540 nm/em: 580 nm) in a spectrofluorometer Victor^2^ Wallac (Perkin Elmer, Turku, Finland). At least two independent experiments were performed in duplicate. Based on the inhibition of cell growth caused by the tested compounds, the percentage of growth inhibition compared to untreated cells was calculated. With these percentages, the concentration that causes cytotoxicity in 50% of macrophages (CC_50_) of macrophages was calculated. Selectivity indices (SI) were calculated, dividing the macrophage CC_50_ by the parasites GI_50_ (SI = CC_50_/GI_50_) [38].

### 2.6. Macrophage-Amastigote Model

We used a previously described methodology [39], with some modifications. Macrophage monolayers, grown in RMPIc medium, for 3 days, were detached from the bottom of the culture chamber, by physical treatment (low temperature and light strokes). Obtained macrophages were seeded in flat-bottom 96-well culture plates, at a density of 2 × 10^5^ cells mL^−1^ (40,000 macrophages/well), in a final volume of 200 µL. Plates were incubated for 2 h, and the supernatant was discarded to remove non-adhered macrophages. Medium was replaced with stationary-phase promastigotes suspended in 200 µL of RPMIc (2 × 10^6^ parasites mL^−1^, 400,000 parasites/well), at a macrophage: parasite ratio 1:10 [40]. The plates were incubated for 4 h. Afterwards the medium was replaced with fresh RPMIc and increasing concentrations of compounds or drugs tested were applied, in volumes of 2 µL, according to the different treatment groups. Plates were kept for 72 h, at 37 °C and 5% CO_2_. The medium was discarded and the wells were washed twice with PBS. Next, a Giemsa staining was performed in situ. It consisted of adding 200 µL of methanol/well, incubating for 5 min, discarding the fixative, and letting it dry at room temperature. Later, the Giemsa solution (10% in PBS *w*/*v*, 200 µL/well) was added, incubated for 10 min, and washed with PBS, allowing it to dry. Finally, the bottom of the plates was detached by percussion and the circular bottoms (wells) were fixed to slides with Permount synthetic resin, for later evaluation (Alcazar W., manuscript in preparation). Cell counting was performed with a 100× immersion objective. A minimum of 200 random macrophages were examined. The percentage of infected macrophages (% infection) and the average number of amastigotes present in infected macrophages (_amastigotes_) were determined. From these data, the infection index (inf-I) was calculated: inf-I = % infection × _amastigotes_. The infection rate represents the number of intracellular amastigotes per 100 macrophages evaluated [40,41]. Additionally, the GI_50_ for the decrease in the infection rate was calculated. At least three independent experiments were performed. The effect of amphotericin-B (AmB) [Sigma-Aldrich, (St. Louis, MO, USA)] was included as positive control of the assay.

### 2.7. Plasma Membrane Potential (ΔΨp)

To evaluate the plasma membrane potential (ΔΨp), the fluorescent compound, bisoxonol, bis[1,3-diethylthiobarbituric acid] trimethine oxonol, was used, as well as a method [41] optimized by Padrón-Nieves et al. [42,43,44]. Initially, calibration curves for each strain, using increasing concentrations of KCl (0.5 to 75 mM), were performed in the late log growth phase *Leishmania* promastigotes in the presence of the potassium ionophore valinomycin. By this procedure, fluorescence values could be transformed into millivolts (mV), applying the Nernst equation, ΔΨp = −59.4 log(120/[KCl]). In 96-well plates, 200-µL aliquots of parasitic suspension were seeded at a cell density of 1 × 10^6^ parasites mL^−1^, dissolved in a solution free of monovalent cations, *N*-methyl-D-glucamine hydrochloride (140 mM NMGHCl; 0.8 mM MgCl_2_; 1 mM CaCl_2_; 10 mM HEPES; 11 mM glucose, pH 7.2). After incubation at RT and in darkness, for 10 min, in the presence of 1 μM of valinomycin, 100 nM bisoxonol was added, and then, increasing concentrations of KCl. After 10 min of incubation in the dark, fluorescence was measured in a Victor^2^ Wallac spectrofluorometer (Perkin Elmer, Turku, Finland), using the appropriate wavelengths (ex: 540 nm/em: 580 nm). This method allowed us also to measure the resting ΔΨp. Once the corresponding calibration curve for each strain was obtained, the effect of compounds on the ΔΨp was evaluated. A similar procedure was followed. Briefly, in 96-well plates, 200-µL aliquots of parasitic suspension were seeded at a cell density of 1 × 10^6^ parasites mL^−1^, dissolved in HEPES solution (HEPESs: 20 mM HEPES, 132 mM NaCl, 3.5 mM KCl, 1 mM CaCl_2_, 0.5 mM MgCl_2,_ pH 7.2) with glucose (5 mM, HEPES-G). After incubation at RT and in darkness, for 15 min, in the presence of test compounds and controls (depolarizers: 1 µM valinomycin, 300 µM cytochalasin B; hyperpolarizers: 500 µM sodium 4,4′-diisothiocyano-2,2′-stilbenedisulfonate, DIDS), 100 nM bisoxonol was added. After 10 min of incubation in the dark, fluorescence was measured, using the appropriate wavelengths (ex: 540 nm/em: 580 nm). At least two independent experiments were performed in duplicate. The treatments were applied in volumes of 2 µL. Valinomycin, a selective ionophore for potassium, and cytochalasin-B, an inhibitor of glucose transport, were used as depolarizing control compounds. DIDS, a voltage-dependent chloride channel inhibitor, was used as a hyperpolarizing control compound. To transform the fluorescence values in millivolts (mV), calibration curves were prepared using increasing concentrations of KCl (0.5, 1.15, 2.5, 5, 10, 20, 40, and 75 mM). This was done for the reference strain LTB0016, as well as for the patient isolates. Finally, to calculate the plasma membrane potential, the Nernst equation was used [ΔΨp = −59.4 log(120/[KCl])] [42,43,44].

### 2.8. Mitochondrial Membrane Potential (ΔΨm)

To evaluate the mitochondrial membrane potential (ΔΨm), the fluorescent compound JC-1 (5,5′,6,6′-tetrachloro-1,1′,3,3′-tetraethylbenzimidazolocarbocyanine iodide) was used, according to a modification of a previously described method [45]. Exponential phase promastigotes were suspended at 26 °C, in RPMIc without phenol red, in a volume of 1 mL, at a cell density of 1 × 10^7^ parasites mL^−1^. The parasites were left to grow for 48 h, in the presence and absence of the test compounds. Then, after centrifuging (500× *g* for 10 min) and washing the parasites twice with PBS, they were resuspended in 200 µL of HEPES-G, and placed in 96-well plates. In the depolarizing control wells, 1 µM carbonyl cyanide 4-(trifluoromethoxy) phenylhydrazone (FCCP) and 20 µM oligomycin were added. The JC-1 probe was then added, at a final concentration of 15 µM. Then, the plates were incubated for 30 min, at RT in the dark. Finally, fluorescence was measured in a Victor^2^ Wallac spectrofluorometer (Perkin Elmer, Turku, Finland) at the appropriate wavelengths (ex: 485 nm/em: 535 and 580 nm). As the mitochondrial membrane is polarized, JC-1 produces intra-mitochondrial aggregates “J-aggregates” that emit a red fluorescence (λ 590 nm). Mitochondrial membrane depolarization induces JC-1 monomeric conformation in the cytoplasm, then the fluorescence turns green (λ 530 nm). The red/green ratio of fluorescence then is an indirect measurement of the mitochondrial membrane potential. A more depolarized mitochondria means a lower ratio of red/green fluorescence.

Relative fluorescence (RF) was calculated, where RF = 580 nm fluorescence/535 nm fluorescence (red/green). A decrease in the value of RF would indicate the collapse of the mitochondrial membrane potential. At least two independent experiments were performed in duplicate. Compounds used as depolarizing controls were: FCCP, an oxidative phosphorylation inhibitor and ionophore, which acts by decoupling the respiratory chain, interrupting the synthesis of ATP by transporting hydrogen ions through the mitochondrial membrane before they can be used as a source of energy; and oligomycin, an antibiotic that inhibits mitochondrial ATPase by binding to the F0 subunit and interfering with the transport of H^+^, thus inhibiting the synthesis of ATP [43].

### 2.9. Nitric Oxide (NO) Production

Following a previously described procedure [46,47], infected and uninfected macrophage cultures were treated with test compounds to analyze their effect in *Leishmania* host cell nitric oxide (NO) production. J774.1 macrophages, at the third day of growth, were collected and seeded in flat-bottom 96-well culture plates, at a density of 1 × 10^6^ cells mL^−1^ (2 × 10^5^ macrophages/well), in 200 µL of RPMIc. The plates were incubated for 2 h; once the supernatant was discarded, the medium was replaced with 200 µL of RPMIc mixed with infectious promastigotes in the stationary phase of growth (1 × 10^7^ parasites/mL, 2 × 10^6^ parasites/well), so that the ratio macrophage: parasites was 1:10 [41]. The plates were then incubated for 4 h, the medium was replaced with fresh RPMIc and the test compounds were applied, according to the different treatment groups. The plates were kept in incubation for 48 h, at 37 °C and 5% CO_2_.

From the culture supernatants, 50-µL aliquots were recovered and transferred to a new 96-well plate to perform the Griess reaction [48], 50 µL of Solution 1 [1% sulfanilamide dissolved in 2.5% phosphoric acid] was added, incubated for 5 min at RT and in the dark. Subsequently, 50 µL of Solution 2 [0.1% *N*-(1-naphthyl) ethylenediamine dihydrochloride dissolved in distilled water] was added, stirred for an additional 5 min, at RT and in the dark. Finally, the colorimetric reading was performed at 550 nm. Three independent experiments were performed in duplicate. The Griess reaction quantifies NO, using nitrite (NO_2_^−^) as the most stable species. NO_2_^−^ concentrations in culture medium reflect indirectly the levels of NO produced by macrophages. Levels of NO_2_^−^ (expressed as µM of NO_2_^−^) were calculated from a calibration curve constructed with increasing concentrations of sodium nitrite (NaNO_2_). MLF (5 µM) and AmB (0.1 µM), known to induce NO production in macrophages, were included as controls.

### 2.10. Superoxide (O_2_^−^) Production

The intracellular oxidative burst was evaluated by the production of the superoxide anion O_2_^−^, using the method of reduction of ferricytochrome c, previously described [46]. Macrophages from preceding experiments were used after removing the supernatant; the cells pretreated with compounds or AmB as positive control of the experiment were resuspended in 100 µL of Hanks saline (HBSS: 137 mM NaCl, 5.4 mM KCl, 1 mM MgSO_4_⋅7H_2_O, 1.3 mM CaCl_2_⋅H_2_O, 0.3 mM Na_2_HPO_4_, 0.44 mM KH_2_PO_4_, 4.2 mM NaHCO_3_, 5 mM glucose, pH 7.2) and incubated for 1 h in the presence of 2 mg mL^−1^ (160 µM) of cytochrome c type III, at 37 °C and 5% CO_2_. The supernatant was saved to measure the change in optical density at 550 nm. Three independent experiments were performed in duplicate. The superoxide anion concentration was calculated using the reduced molar extinction coefficient of cytochrome c, 21 mM^−1^ cm^−1^. Therefore, [O_2_^−^] = (ΔOD/6.3) × 100, where ΔOD is the variation of the optical density (reading-blank), and the result obtained corresponds to the concentration of superoxide anion expressed in nanomoles of O_2_^−^ released/2 × 10^5^ cells. MLF (5 µM) and amphotericin-B (AmB, 0.1 µM), known to induce NO production in macrophages, were included as controls.

### 2.11. Statistics

GraphPad Prism 8 software (2018, San Diego, CA, USA) was used. The Kolmogorov–Smirnov normality test and the Barlett test for homogeneity of variances, with a 95% confidence, were used to test the null hypothesis that the data were taken from a Gaussian distribution. Since the value of *p* > 0.05 showed that the data did not deviate from the normal distribution, the variance analysis and the Bonferroni multiple comparisons test were used to compare the results between different test conditions [49]. Results were expressed as mean ± standard error of the mean. In all tests, a value of *p* < 0.05 was considered statistically significant. GI_50_ and CC_50_ values were calculated by non-linear regression of the sigmoid response dose curve with variable slope [50]. Supplementary Materials III includes tables of the statistical analysis performed for each of the methods used to analyze the potential mechanism of action of compounds.

## 3. Results

### 3.1. Selection of the Tool Compounds

To select compounds that may be interesting for their potential anti-leishmanial activity, 20 structurally simple (compounds **1**–**20**) and 8 heterocyclic (compounds **21**–**28**) betulin derivatives [28,29] were evaluated by their capacity to inhibit reference strain (LTB0016) growth at one sole concentration of 50 µM (upper limit to consider their clinical value) [38]. For details on their chemical formula, structure, and molecular weight, please consult Supplementary Materials I.

A total of eight compounds that reduced at least 75% of parasite growth were forwarded to the next step of analysis as “active compounds”. These compounds were tested at increasing concentrations to find out their GI_50_ against the *Leishmania* reference strain.

Three simple betulin derivatives (**1**, **17**, and **18**) reduced parasite growth to less than 25%, with **18** being the most active compound, reducing promastigote growth to 3.32% compared to untreated cells. Five heterocyclic betulin derivatives (**22**, **24**, **25**, **26**, **27**) reduced parasite growth to less than 25%, with **26** being the most active compound, reducing promastigotes growth to 4.28%.

To quantify the leishmanicidal effect of the best compounds of both groups of betulin derivatives, GI_50_ was determined in promastigotes of LTB0016, at increasing concentrations from 1 to 100 µM and 72 h of incubation (see Table 1).

One-way ANOVA analysis, followed by Bonferroni test, allowed identification of significant differences (*p* < 0.05) of the tested compounds vs. SbIII [GI_50_ = 5.51 (IL = 4.92–SL = 6.08 µM] against promastigotes. Compounds **17**, **18**, **22**, **26**, and **27** were as effective as the reference compound to inhibit LTB0016 growth. However, a significant difference against SbIII (*p* = 0.0413) was only detected for **18**, being even stronger than it. Potential cytotoxicity was evaluated in J774.1 macrophages. Due to their low cytotoxicity, **18** and **22** presented the best SI (SI > 15) and were forwarded to the next step of research.

### 3.2. Effect of Compounds on Intracellular Amastigotes

To confirm the anti-leishmanial effect of the compounds **18** and **22**, GI_50_ was determined for intracellular amastigotes of reference strain LTB0016 and patient isolates, using the amastigote-macrophage model. Thus, infected macrophages were exposed to increasing concentrations of **18** and **22** (0.1, 0.5, 1, 5, and 10 µM). The infectious index data is included as Supplementary Materials (Supplementary Materials II). Final results are summarized in Table 2. Compounds **18** and **22** showed GI_50_ < 1 µM and can be considered safe in their toxicity against J774.1 macrophages (SI > 20), both for reference strain LTB0016 and patient isolates.

### 3.3. In the Search of the Mechanism of Action of Betulin Derivatives

In the search of the mechanism of action of the selected compounds, their effect on the plasma membrane potential and mitochondrial membrane potential was evaluated in promastigotes. In non-infected and infected macrophages, the effect of selected compounds was evaluated on nitric oxide production and on superoxide production.

#### 3.3.1. Plasma Membrane Potential (ΔΨp) in Promastigotes

To evaluate the effect of the most promising compounds on the plasma membrane potential (ΔΨp), promastigotes were exposed during 15 min to **18** and **22**. Selected concentrations were their GI_50_ and twice GI_50_ (**18**, 1 and 2 µM; **22**, 2 and 4 µM). Untreated cells, as well as cells treated with depolarizing molecules (valinomycin, 1 µM; cytochalasin B, 300 µM) and one hyperpolarizing compound (DIDS, 500 µM) were included. As the depolarizing probe, the lipophilic anion bisoxonol [100 nM] was used.

The resting ΔΨp of parasites was then established (LTB0016: −181.97 ± 19 mV; VE98MR = −166.75 ± 11 mV and VE2000MM = −158.30 ± 8 mV). The isolates presented more depolarized resting membrane potentials than the reference strain (see Supplementary Materials III for statistics).

**Figure 1:**
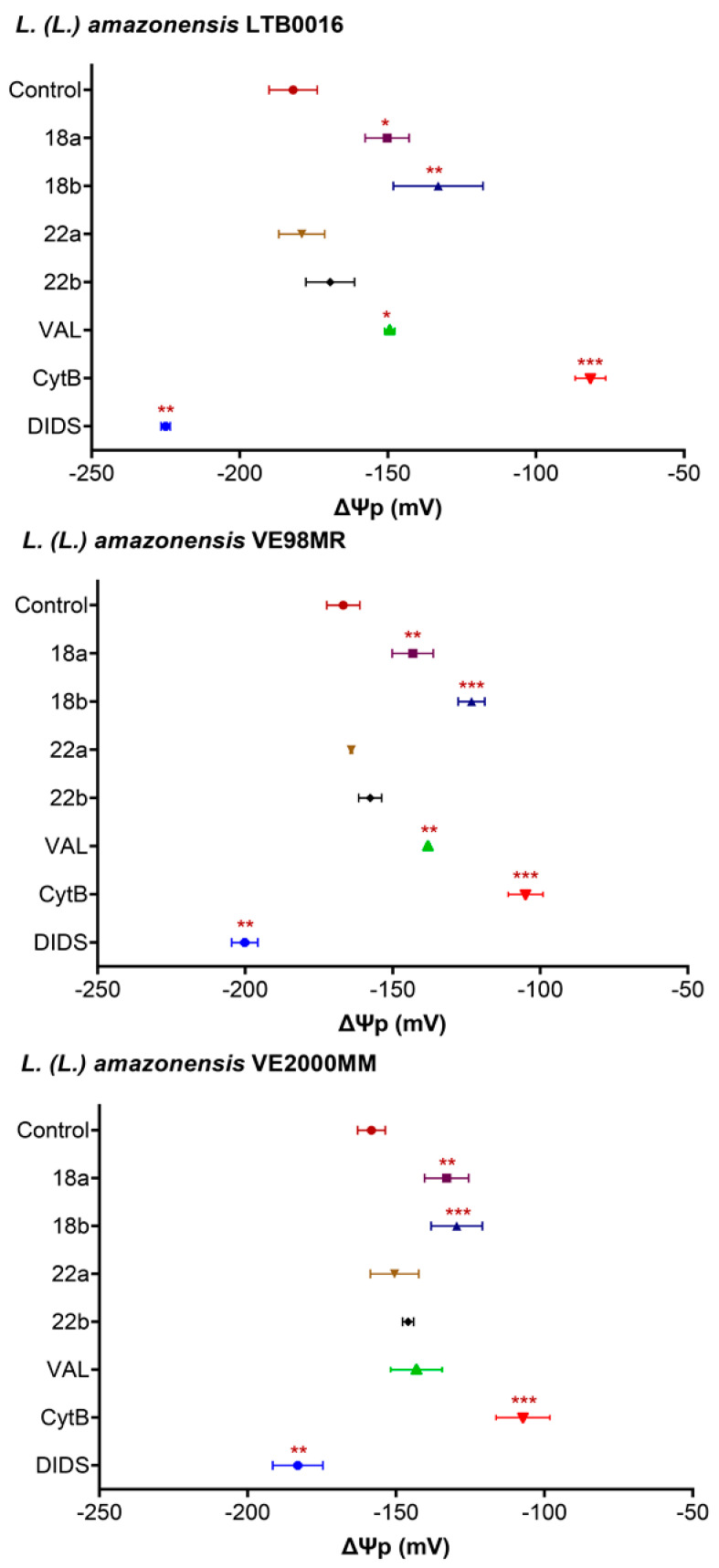
Effect of betulin derivatives on plasma membrane potential (ΔΨp) in *L. (L.) amazonensis* reference strain LTB0016 and isolates VE98MR and VE2000MM promastigotes isolated from patients, at two increasing concentrations (a and b): **18** (1 and 2 µM), **22** (2 and 4 µM), incubated for 15 min. Each bar or point represents the average (*n* = 3 to 6 independent experiments) with its standard error (SEM). * *p* < 0.05; ** *p* < 0.005; *** *p* < 0.0005 compared to control without treatment. Control: without treatment; VAL: valinomycin, 1 µM; CytB: cytochalasin B, 300 µM; DIDS: sodium 4,4’-diisothiocyano-2,2’-stilbenedisulfonate, 500 µM.

Figure 1 demonstrates that only **18** was able to significantly depolarize the ΔΨp in all evaluated strains when compared to untreated controls (*p* < 0.05). The effect was similar to that depicted by valinomycin and concentration dependent, except in the case of VE2000MM. This strain was not sensitive to valinomycin.

#### 3.3.2. Mitochondrial Membrane Potential (ΔΨm) in Promastigotes

To evaluate if the effect of the betulin derivatives on parasites was related to their action on mitochondria, or related to programmed cell death, their effect on the mitochondrial membrane potential (ΔΨm) was evaluated. Promastigotes were exposed to two concentrations of the compounds, equivalent to their GI_50_ and twice GI_50_ (as before, **18**: 1 and 2 µM; **22**: 2 and 4 µM), during 48 h. As controls, untreated cells, as well as cells treated with depolarizing compounds (FCCP 1 µM and oligomycin 20 µM) were included. As the label for ΔΨm, the lipophilic cationic probe JC-1 was used.

**Figure 2:**
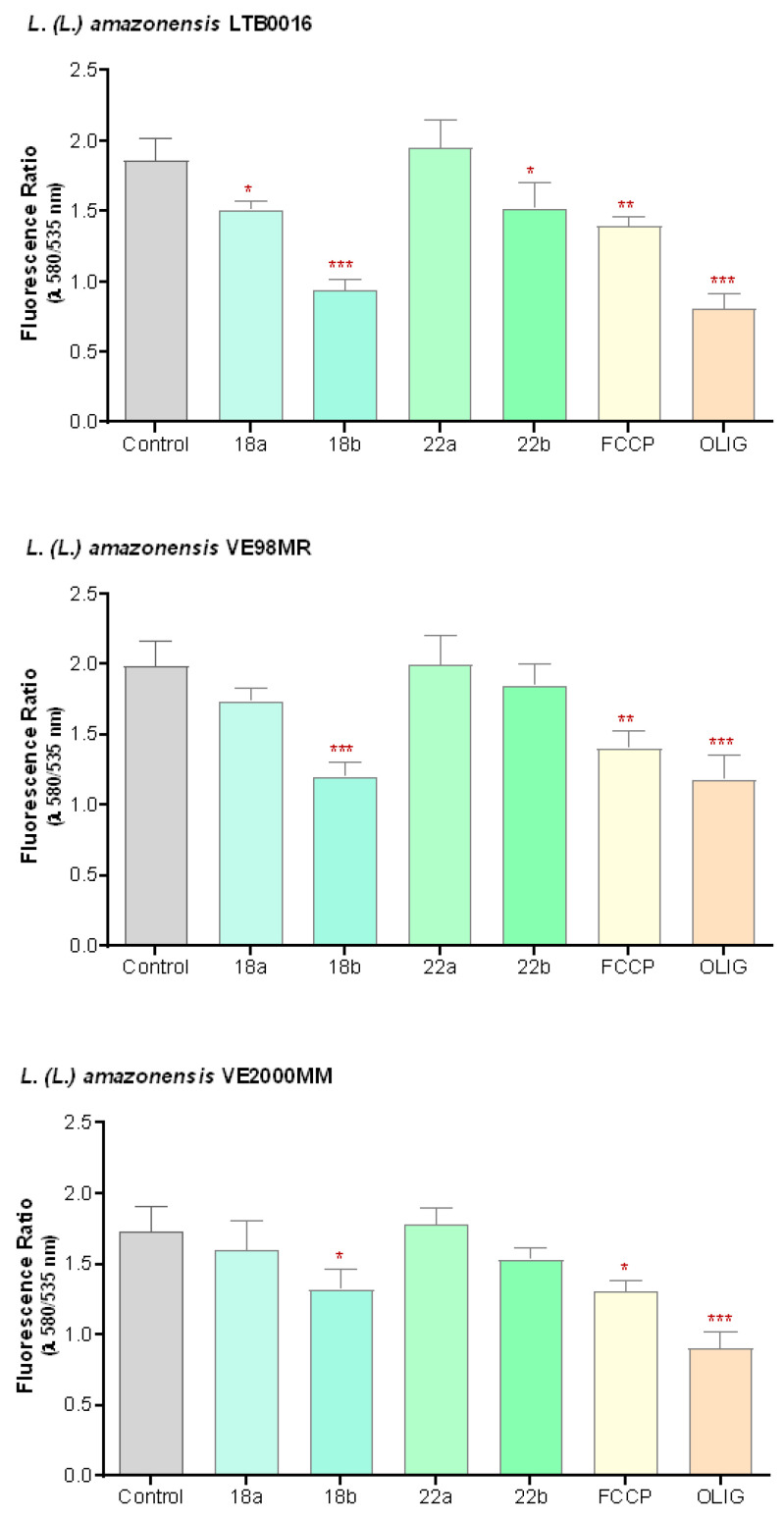
Effect of betulin derivatives on mitochondrial membrane potential (ΔΨm) in *L. (L.) amazonensis* reference strain LTB0016 and isolates VE98MR and VE2000MM promastigotes isolated from patients, at two increasing concentrations (a and b): **18** (1 and 2 µM), **22** (2 and 4 µM), incubated for 48 h. Each bar represents the average (*n* = 3 independent experiments in duplicate) with its respective standard error (SEM). * *p* < 0.05; ** *p* < 0.005; *** *p* < 0.0005 with respect to the control without treatment. Control: untreated control; FCCP: carbonyl cyanide 4-(trifluoromethoxy) phenylhydrazone, 1 µM; OLIG: oligomycin, 20 µM.

Figure 2 demonstrates how **18** depolarizes ΔΨm in a concentration-dependent way when compared to untreated cells (*p* < 0.05) in all studied cells. The effect of **18** at its higher concentration (2 µM) in LTB0016 and VE98MR was comparable to that of oligomycin 20 µM, a compound that quickly inhibits the mitochondrial ATPase and ATP synthesis. **22** also inhibited ΔΨm, and a significant difference (*p* = 0.025) was only present at the highest concentration used (4 µM) and for LTB0016 (see Supplementary Materials III for statistical details).

#### 3.3.3. Nitric Oxide Production (NO) in Macrophages

**Figure 3:**
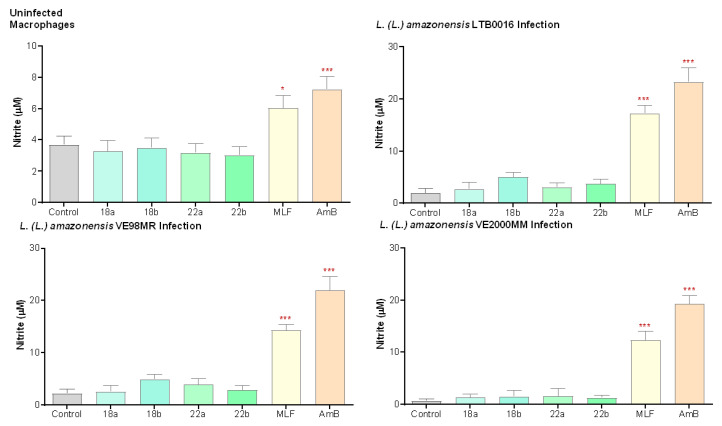
Effect of betulin derivatives on the production of nitric oxide (as µM nitrite) by healthy macrophages or macrophages infected either with LTB0016, 98MR, or VE2000MM, at two increasing concentrations (a and b): **18** (1 and 2 µM), **22** (2 and 4 µM), incubated for 48 h. Each bar represents the average (*n* = 3 independent experiments) with its respective standard error (SEM). * *p* < 0.05; *** *p* < 0.0005 with respect to the control without treatment. Control: untreated control; MLF: miltefosine, 5 µM; AmB: amphotericin B, 0.1 µM.

To evaluate if the anti-leishmanial activity of compounds **18** and **22** is related to modulating effects on the microbicidal activity of macrophages, non-infected and infected cultured macrophages were exposed to two concentrations of the compounds, promastigote GI_50_ and twice GI_50_ (**18**: 1 and 2 µM; **22**: 2 and 4 µM). The NO production was indirectly determined through the evaluation of nitrite (NO_2_^−^) levels by the Griess reaction. Compounds **18** and **22** were not capable of inducing NO production in any of the strains tested (Figure 3). Supplementary Materials III details the associated statistics.

#### 3.3.4. Superoxide Production (O_2_^−^) in Macrophages

**Figure 4:**
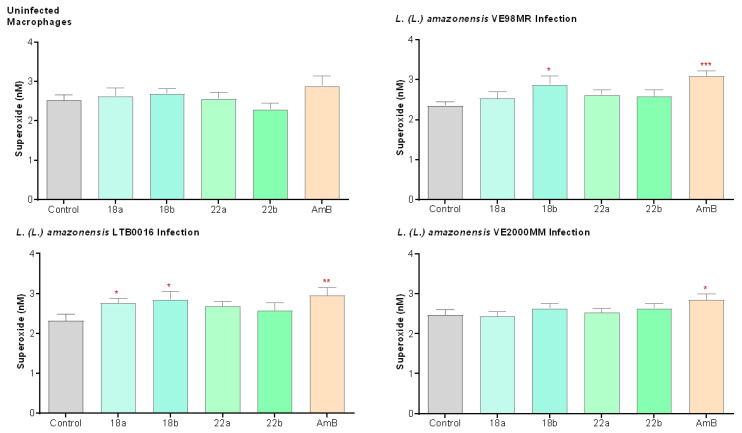
Effect of betulin derivatives on the production of superoxide anion (O_2_^−^) by healthy macrophages, or macrophages infected by LTB0016, VE98MR, or VE2000MM, at two increasing concentrations (a and b): **18** (1 and 2 µM), **22** (2 and 4 µM), incubated for 48 h. Each bar represents the average (*n* = 3 independent experiments) with its respective standard error (SEM). * *p* < 0.05; ** *p* < 0.005; *** *p* < 0.0005 with respect to the control without treatment. Control: control without treatment; AmB: amphotericin, 0.1 µM.

Additionally, the O_2_^−^ was evaluated in healthy macrophages, as well as in macrophages infected either with the reference strain LTB0016 or with the patient isolates VE98MR or VE2000MM and treated for 48 h with **18** and **22** (**18**: 1 and 2 µM; **22**: 2 and 4 µM; respectively). Cells were further incubated for 1 h in the presence of cytochrome C-III (CYT-CIII 160 µM). By using the method of cytochrome reduction and the molar extinction coefficient of CYT-CIII, superoxide production, expressed in nanomoles of O_2_^−^ released by 2 × 10^5^ cells, was determined. In healthy macrophages, betulin derivatives did not induce superoxide production. However, in LTB0016- and VE98MR-infected macrophages, the betulin derivative **18** induced superoxide production (Figure 4). Supplementary Materials III details the associated statistics.

## 4. Discussion

Pentacyclic triterpenes like betulin and lup-20(29)-ene-3ß,28-diol, are isolated from birch outer bark (*Betula* spp. Betulaceae) with a yield around 30% of dry matter [51]. Derivatives from the original active betulin compound can be chemically modified at the hydroxyl group C-3, the double bond C-20, and the primary hydroxyl C-28. Betulin derivatives with anti-leishmanial properties have been described previously [27,28,29,30].

Although plant extracts and compounds against *L. (L.) amazonensis* [52,53,54,55] have been thoroughly evaluated, the betulin derivatives herein described have been tested against New World *Leishmania* spp. but not against patient isolates with therapeutic failure. In the present work, two *L. (L.) amazonensis* Venezuelan isolates, obtained from patients with therapeutic failure against SbV, were used to characterize the activity of betulin derivatives.

Here, 28 semisynthetic betulin derivatives selected according to Alakurtti et al. [29,55] were tested at 50 µM. Eight compounds showed an anti-leishmanial effect (**1**, **17**, **18**, **22**, **24**, **25**, **26**, and **27**), inhibiting *L. (L.) amazonensis* growth by at least 75%. Previously, we demonstrated that derivatives **14**, **17**, **18**, **25**, **26**, and **27** were active at less than 10 µM in *L. (L.) braziliensis,* reference strain MHOM/BR/LTB300, and that **18**, **26**, and **27** inhibited parasite migration at nano and picomolar concentrations, suggesting a chemotactic effect of betulin derivatives that might be vital for host–parasite interaction [28].

The corresponding promastigote GI_50_ against the reference compounds (AmB, PNT, SbIII, MLF) were defined. As pentavalent antimonials (SbV) constitute pro-drugs that need biological reduction to their trivalent form (SbIII) to be leishmanicidal [56], we used the latter as potassium antimonyl tartrate trihydrate instead of the pentavalent form, meglumine antimoniate.

Promastigotes of Venezuelan isolates herein analyzed were less susceptible to SbIII (VE98MR, GI_50_ = 9.08 µM; VE2000MM, GI_50_ = 13.60 µM) than the reference strain (LTB0016 GI_50_ = 5.51 µM). Additionally, VE2000MM was slightly less susceptible to MLF (GI_50_ = 5.13 µM) than the reference strain (LTB0016 GI_50_ = 4.17 µM). These results suggest that VE98MR, and specifically VE2000MM, might be parasites refractive to conventional treatments, thus being suitable isolates for in vitro evaluation of new anti-leishmanial compounds.

Data not detailed in this manuscript suggest that the isolates’ susceptibility against NaNO_2_ was such that GI_50_ was four-fold higher (VE98MR, GI_50_ = 3.28 µM; VE2000MM, GI_50_ = 4.79 µM) than the reference strain (LTB0016, GI_50_ = 0.91 µM), thus suggesting that the *Leishmania* isolates, obtained from patients suffering therapeutic failure against meglumine antimoniate, can be classified as chemoresistant as has been described for antimonial-resistant *L. (L.) infantum* isolated from Brazilian patients with therapeutic failure [57]. Additionally, the GI_50_ against K_2_Cr_2_O_7_ (>2.5 mM) for both isolates was significantly higher than that obtained for the reference strain (LTB0016 GI_50_ = 1.06 mM); similar results have been published for SbV-resistant *L. (L.) donovani* tolerant to K_2_Cr_2_O_7_ [58].

As compounds **18** and **22** [25,59] were classified as effective and relatively secure (GI_50_ ≤ 2 µM, cytotoxic CC_50_ > 45 µM, SI > 20), they were furthered to explore their mechanism of action.

In the amastigote-macrophage model, the isolates (GI_50_: VE98MR = 1.76 µM, VE2000MM = 0.72 μM) were less susceptible to **18** than the reference strain LTB0016 (GI_50_: = 0.20 µM). Similar results were obtained for **22**; the less susceptible isolate, VE2000MM, demonstrated a GI_50_ of 1.69 µM, and the reference strain of 0.21 µM. VE98MR and VE2000MM might be less susceptible due to potential cross-resistance mechanisms between SbV and betulin derivatives as more than 3-fold doses were needed to reach similar effects in isolates vs. the reference strain.

These concentrations did not affect macrophages’ viability (SI > 20), suggesting that **18** and **22** are worthy of being analyzed as potential candidates against parasites refractory to conventional treatment, as has been tested against *L. (L.) donovani* susceptible to SbV or selected as resistant to the same compound [60], as well as against *Plasmodium falciparum* (clone W2), resistant to chloroquine [61] for which betulin derivatives like *O*-disuccinoyl betulin, betulonic acid, and other betulinic acid derivatives have proved to be effective.

Our results suggest that GI_50_ values for **18** in *L. (L.) amazonensis* LTB0016 (GI_50_ promastigote = 0.90 µM; GI_50_ amastigote = 0.20 µM) were lower than those described for the same compound in *L. (L.) donovani* axenic amastigotes (GI_50_ = 22.80 µM) [29]. Differences may respond to species-specific internal factors, with *L. (L.) donovani* being intrinsically less susceptible to **18** than *L. (L.) amazonensis.* Alternatively, the used parasitic stage, axenic amastigotes vs. intracellular amastigotes, may be influencing the obtained results.

Our data suggest that the carboxyl group at position C28 is not indispensable for betulin anti-leishmanial activity, as suggested for *L. (L.) major* [62]. For example, **11** (betulinic acid), at 50 µM, inhibited *L. (L.) amazonensis* LTB0016 growth by 44%; other carboxyl compounds, (**13** and **14**) did not show enough anti-leishmanial activity.

In *L. (V.) braziliensis* promastigotes, the most potent compound was **26** (3,28-diformylbetulin triazolodione) with a GI_50_ < 1 µM [29]. Likewise, a betulin aldehyde derivative reduced infection up to 88% at 136 µM and 58% at 68 µM of intracellular *L. (L.) amazonensis* amastigotes [63]. Herein, **26** was also within the initially selected compounds. However, as previously mentioned, only **18** and **22** showed significant differences that made them worthy of further analysis. These results support our data regarding the moderate activity identified for betulinic aldehyde (**12**) that at 50 µM inhibited *L. (L.) amazonensis* growth by 67% and suggest that there might be species variances that could explain the differences herein found when compared to previous data in *L. (V.) braziliensis.*

Compound **22** is a heterocyclic adduct between 3,28-di-*O*-acetyllupa-12,18-diene and 4-ethylurazine, while the structurally related **25** is another heterocyclic adduct between 3,28-di-*O*-acetyllupa-12,18-diene and 4-methylurazine [55]. Compound **25**, although cytotoxic at all evaluated concentrations, is an effective derivative with a GI_50_ of 8.9 µM, in *L. (L.) donovani* axenic amastigotes. On the other hand, **22**, was slightly toxic for J774.1 macrophages (CC_50_ = 61.13 µM) with minimal GI_50_ against *L. (L.) amazonensis* intracellular amastigotes = 0.21 µM), and very high selectivity index (SI = 291). Heterocyclic derivatives bulkier in C3, C28, or N4 were less active.

The anti-leishmanial activity of betulin derivatives associates in Old World *Leishmania* with inhibitory effects over enzymes fundamental for parasite survival, such as topoisomerase IB [64] and trypanothione synthetase, TryS [65] as well as on the promoted parasite oxidative stress that ends up in programmed cell death [66]. However, specific targets and molecular mechanisms involved in their action still are not completely understood [67], and for New World species are not known at all. Herein, we explored their mechanism of action.

In organisms like protozoa, bacteria, pathogenic fungi, and tumor cells, betulin derivatives’ target seems to be located at the plasma membrane [68]. Examples also exist in *Leishmania* that illustrate the effect of compounds on the membrane, specifically affecting the ΔΨp, i.e., AmB in *L. (L.) infantum* [69], clomipramine and imipramine in *L. (L.) donovani* and *L. (L.) major* [70] or peptides like cecropin-A and melittin in *L. (L.) donovani* [71], or lupeol, an anti-leishmanial triterpene from *Sterculia villosa* for *L. (L.) infantum* [72], and *L. (L.) donovani* [73]. In this line of evidence, we report that betulin derivative **18**, induced, in a very short time (15 min), the collapse of the plasma membrane potential (ΔΨp) in a concentration-dependent way. The effect was comparable to that imposed by valinomycin (1 µM) and cytochalasin B (300 µM).

On the other hand, betulin **1** and betulin derivatives induce programmed cell death in *L. (L.) donovani* promastigotes [66] and the betulin derivative *O*-disuccinoyl betulin induces non-dependent caspase apoptosis [64]. The effect of **1** in *L. (L.) donovani* correlates with the inhibition of TryS [65], resulting in intracellular redox homeostasis loss [66].

Besides that, medicinal plants and their secondary metabolites may have immunomodulatory properties [74] [superoxide (O_2_^−^) and nitric oxide (NO) production], potentially useful against leishmaniasis [75]. *L. major* amastigote elimination depends preferentially on NO production [75] and *L. (V.) braziliensis* abolition seems to strictly depend on both O_2_^−^ and ROS generation [76]. Of note, in macrophages infected with *L. (L.) donovani*, lupeol induced NO production and increased the production of proinflammatory cytokines, decreasing the production of anti-inflammatory cytokines [73].

Nitric oxide is essential for macrophage activation and is produced in *Leishmania*-infected macrophages [77,78]; additionally, metacyclic *L. amazonensis* promastigotes produce NO and axenic amastigotes produce NO levels 10-fold higher than those produced by metacyclic promastigotes as has been described by Acuña and collaborators (2017) [79] and references within. However, similar to what we herein described in both the reference strain and patient isolates and in *L. amazonensis*-infected macrophages, there is a lower production of NO than in un-infected macrophages. *L. amazonensis* seems to have an adaptive strategy to escape from host defense. This strategy seems to be produced by the activation of the NF-κB repressor complex p50/p50, a specific host transcriptional response that negatively regulates the expression of inducible nitric oxide synthase. This feature seems to favor the establishment and success of *L. amazonensis* infection [80].

In our hands, **18** and **22** were not capable of inducing NO production for any of the strains tested. However, compound **18** seems to influence the oxidative response of J774.1 macrophages infected with *L. (L.) amazonensis* LTB0016 and VE98MR, inducing an oxidative burst and thus increasing O_2_^−^ production.

Considering the structural similarity between lupeol and betulin derivatives, both terpenoids may be addressing the same cellular target. Molecular docking analysis [73] suggests that these compounds may be capable of attachment to the parasite surface protein GP63, thus affecting the cells’ signaling processes that lead to macrophage activation [81]. On the other hand, an in silico analysis of a 58-member library of betulin derivatives (including **18**) identified the membrane transport protein D1 as a potential target for this compound in *L. (L.) donovani* [67].

Finally, whether molecular functional events associated with the production of non-caspase-dependent apoptosis in *L. (L.) donovani* [64] and produced by *O*-disuccinoyl betulin [66] or inhibition of TryS [65]) are affected by betulin derivatives like compound **18** in *L*. (*L*.) *amazonensis* has to be further analyzed.

Altogether, our results suggest that diverse targets might be addressed by **18** in *L. (L.) amazonensis*. Therefore, further experiments are needed to fully elucidate the final mechanism of action of this compound.

## Figures and Tables

**Table 1:** In vitro activity, toxicity, and selectivity indices of the best betulin derivatives against the reference strain LTB0016.

Betulin Derivatives	Parasite Growth % [50 µM]	Promastigote (GI_50_)			Macrophages J774.1 (CC_50_)			SI
		$\bar{X}$_(µ__M)_	IL	SL	$\bar{X}$_(µ__M)_	IL	SL	CC_50_/GI_50_
**1** ^1^	19.76	19.33	14.14	24.53	25.08	8.95	41.22	1.30
**17** ^1^	6.49	1.35	−0.03	2.74	19.26	10.10	28.41	14.23
**18** ^1^	3.32	0.90	0.24	1.55	48.09	33.17	63.01	53.63
**22** ^2^	7.98	1.98	0.99	2.98	61.13	52.91	69.35	30.82
**24** ^2^	10.26	9.05	6.95	11.15	51.06	34.78	67.34	5.64
**25** ^2^	11.70	6.46	2.63	10.30	65.93	51.76	80.10	10.20
**26** ^2^	4.28	3.85	−0.23	7.93	21.86	15.72	28.00	5.68
**27** ^2^	7.34	4.23	0.42	8.04	21.12	16.86	25.38	4.99
SbIII	ND	5.51	4.92	6.08	ND	ND	ND	ND
AmB	ND	0.068	0.05	0.08	ND	ND	ND	ND
PNT	ND	0.93	0.51	1.35	ND	ND	ND	ND
MLF	ND	4.17	3.79	4.54	ND	ND	ND	ND

LTB0016 promastigotes and J774.1 macrophages were exposed to increasing concentrations (1, 5, 10, 50, and 100 µM) of the compounds for 72 h. Summarized data are expressed as the mean concentration ($\bar{X}$) of three independent experiments performed in duplicate, including 95% confidence interval (IL: inferior limit, SL: superior limit). GI_50_ and CC_50_ values were determined by lineal regression of dose response curves with variable slope, using the GraphPad Prism 8/2018. GI_50_: 50% inhibitory growth concentration, CC_50_: 50% cytotoxic concentration, SI: selectivity index. ^1^ Simple betulins, ^2^ heterocyclic betulins. SbIII was used as control to evaluate the susceptibility of extracellular parasites, since it is well described that *Leishmania* promastigotes are not susceptible to SbV. AmB, PNT, and MLF were used as control commercial drugs to compare the susceptibility of promastigotes.

**Table 2:** In vitro activity of compounds against intracellular amastigotes of the reference strain LTB0016 and patient isolates VE98MR and VE2000MM.

	GI_50_ (µM)			MØ J774.1	SI (CC_50_/GI_50_)		
	LTB0016	VE98MR	VE2000MM	CC_50_^1^ (µM)	LTB0016	VE98MR	VE2000MM
**18**	0.20	1.76	0.72	48.09	240.45	27.32	66.79
**22**	0.21	0.63	1.69	61.13	291.10	97.03	36.17
**AmB**	0.020	0.073	0.080	>10	>500	>136	>125

LTB0016, VE98MR, and VE2000MM intracellular amastigotes and J774.1 macrophages were exposed to increasing concentrations of the control drug AmB or experimental compounds **18** and **22** for 72 h. Data are presented as mean values of three independent experiments. GI_50_ and CC_50_ values were determined by lineal regression of dose–response curves using GrapPad Prism 8/2018. GI_50_: 50% inhibitory growth concentration, CC_50_: 50% cytotoxic concentration, SI: selectivity index. ^1^ The CC_50_ values for compounds **18** and **22** come from Table 1.

## Data Availability

Not applicable.

## Supplementary Materials

The following are available online at https://www.mdpi.com/2076-2607/9/2/320/s1.
