# Supplementary material for: Leishmanicidal Activity of Betulin Derivatives in Leishmania amazonensis; Effect on Plasma and Mitochondrial Membrane Potential, and Macrophage Nitric Oxide and Superoxide Production
Source: Microorganisms. 2021 Feb 4;9(2):320. doi: 10.3390/microorganisms9020320 (PMC7913927; doi:10.3390/microorganisms9020320)
Supplement: Supplementary file 1 [file microorganisms-09-00320-s001.zip › Alcazar et al. 2020_Supplementary Material I-III/Alcazar et al. 2020 Sup Mat I.pdf]

Simple betulin derivatives

Compound	Chemical formula	Molecular weight (g/mol)	Chemical structure
**1** (betulin)	$C_{30}H_{50}O_2$	442.7	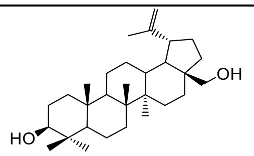
**2**	$C_{30}H_{52}O_2$	444.8	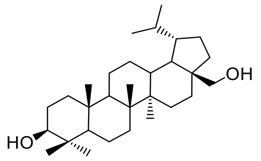
**3**	$C_{32}H_{52}O_3$	484.8	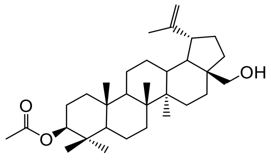
**4**	$C_{34}H_{54}O_4$	526.8	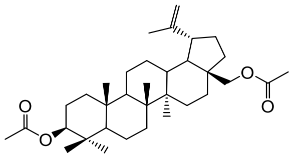
**5**	$C_{36}H_{60}O_4$	568.9	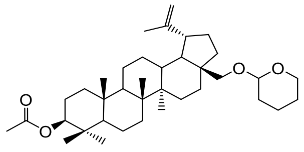
**6**	$C_{40}H_{62}O_6$	638.9	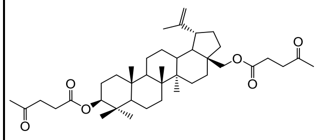
**7**	$C_{35}H_{58}O_3$	526.9	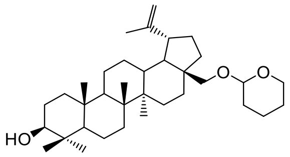
**8**	$C_{39}H_{56}O_3$	572.8	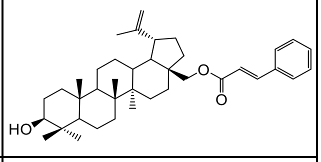
**9**	$C_{36}H_{53}NO_3$	547.8	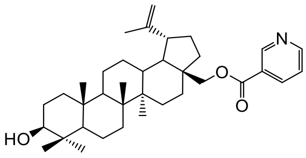
**10**	$C_{40}H_{64}O_3$	592.9	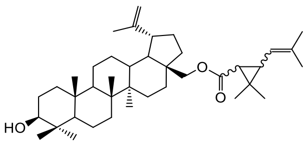

**11**	$C_{30}H_{48}O_3$	456.7	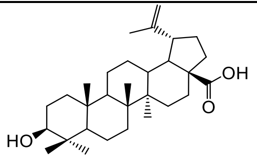
**12**	$C_{30}H_{46}O_2$	438.7	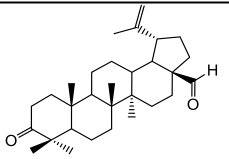
**13**	$C_{30}H_{48}O_3$	456.7	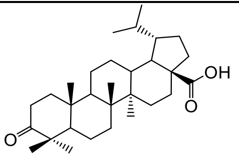
**14**	$C_{30}H_{46}O_3$	454.7	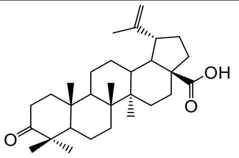
**15**	$C_{38}H_{52}O_5$	588.4	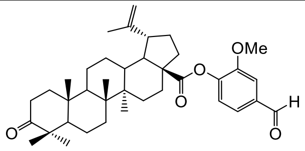
**16**	$C_{36}H_{55}NO_6$	597.8	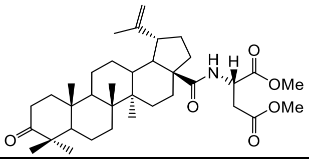
**17**	$C_{30}H_{48}N_2O_2$	468.7	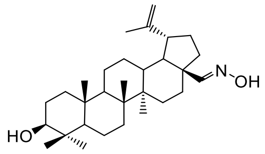
**18**	$C_{30}H_{49}NO_2$	455.7	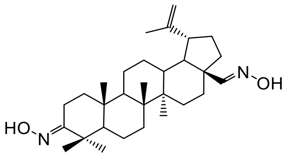
**19**	$C_{32}H_{52}O_3$	484.8	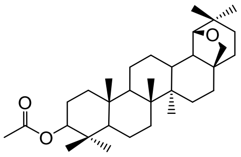
**20**	$C_{30}H_{46}O_3$	454.7	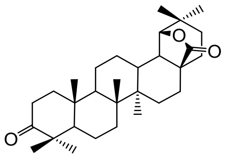

Heterocyclic betulin derivatives

**21**	$C_{42}H_{57}N_3O_6$	699.9	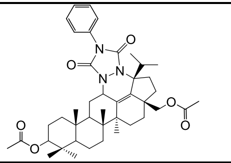

**22**	$C_{38}H_{57}N_3O_6$	651.9	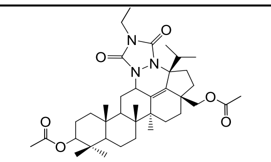
**23**	$C_{41}H_{63}N_3O_6$	694.0	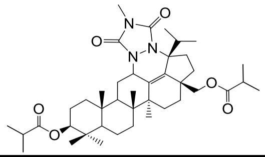
**24**	$C_{39}H_{59}N_3O_6$	665.9	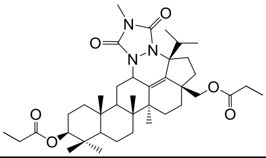
**25**	$C_{37}H_{55}N_3O$	637.9	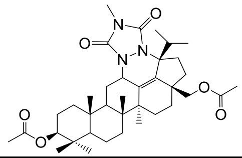
**26**	$C_{35}H_{51}N_3O_6$	609.8	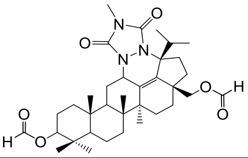
**27**	$C_{33}H_{49}N_3O_4$	551.8	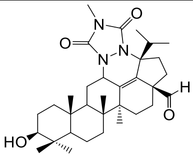
**28**	$C_{33}H_{47}N_3O_4$	549.8	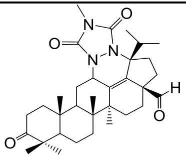
