# Supplementary material for: Leishmanicidal Activity of Betulin Derivatives in Leishmania amazonensis; Effect on Plasma and Mitochondrial Membrane Potential, and Macrophage Nitric Oxide and Superoxide Production
Source: Microorganisms. 2021 Feb 4;9(2):320. doi: 10.3390/microorganisms9020320 (PMC7913927; doi:10.3390/microorganisms9020320)
Supplement: Supplementary file 1 [file microorganisms-09-00320-s001.zip › Alcazar et al. 2020_Supplementary Material I-III/Alcazar et al. 2020 Sup Mat III.docx]

**EFFECT OF LEADING COMPOUNDS ON PLASMA MEMBRANE POTENTIAL (ΔΨp)**

**Effect of leading compounds on plasma membrane potential (ΔΨp) in reference *Leishmania amazonensis* LTB0016 promastigotes.**


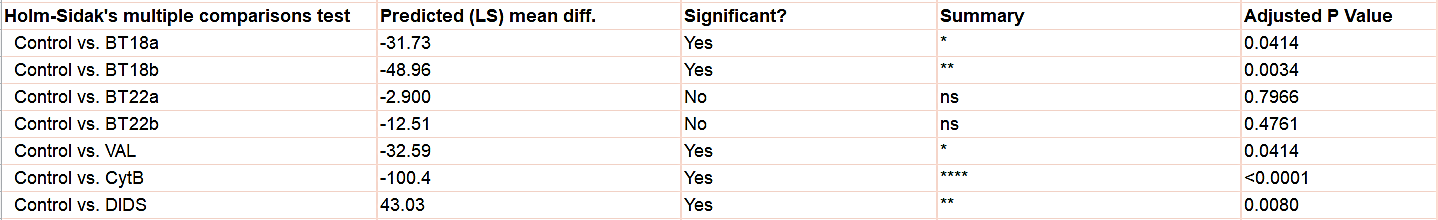


*Test was performed using GraphPad Prism version 8.0.2/2019 for Windows.*

**Effect of leading compounds on plasma membrane potential (ΔΨp) in *Leishmania amazonensis* VE98MR promastigotes isolated from patient.**


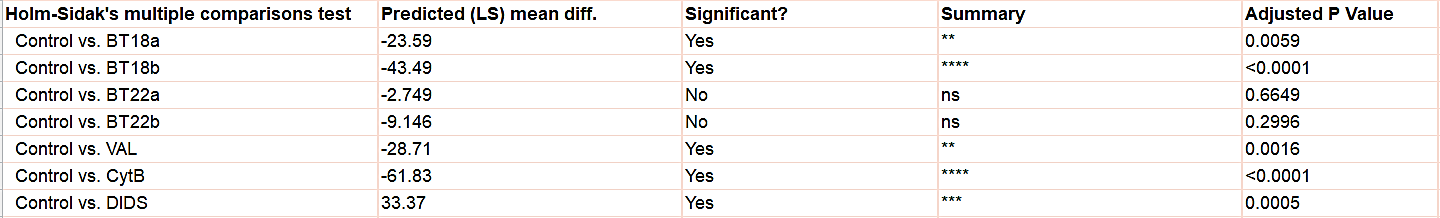


*Test was performed using GraphPad Prism version 8.0.2/2019 for Windows.*

**Effect of leading compounds on plasma membrane potential (ΔΨp) in *Leishmania amazonensis* VE2000MM promastigotes isolated from patient.**


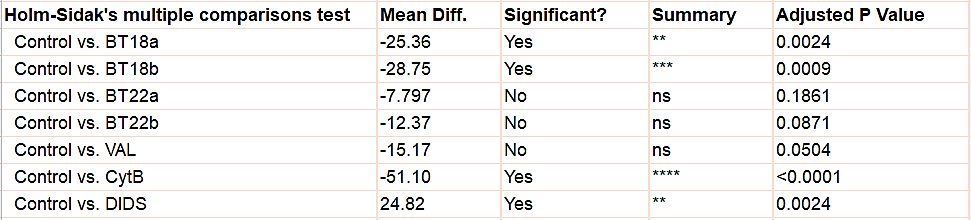


*Test was performed using GraphPad Prism version 8.0.2/2019 for Windows.*

**EFFECT OF LEADING COMPOUNDS ON MITOCHONDRIAL MEMBRANE POTENTIAL (ΔΨm)**

**Effect of leading compounds on mitochondrial membrane potential (ΔΨm) in reference *Leishmania amazonensis* LTB0016 promastigotes.**


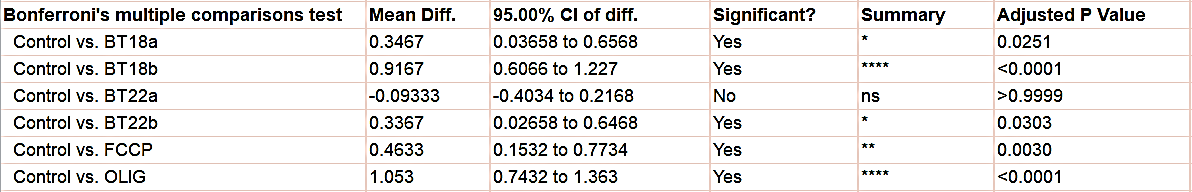


*Test was performed using GraphPad Prism version 8.0.2/2019 for Windows.*

**Effect of leading compounds on mitochondrial membrane potential (ΔΨm) in *Leishmania amazonensis* VE98MR promastigotes isolated from patient.**


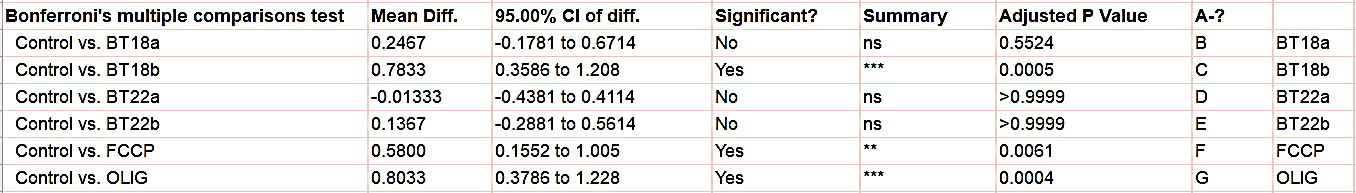


*Test was performed using GraphPad Prism version 8.0.2/2019 for Windows.*

**Effect of leading compounds on mitochondrial membrane potential (ΔΨm) in *Leishmania amazonensis* VE2000MM promastigotes isolated from patient.**


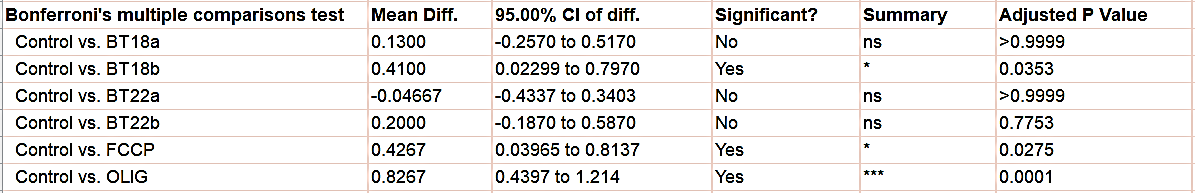


*Test was performed using GraphPad Prism version 8.0.2/2019 for Windows.*

**EFFECT OF LEADING COMPOUNDS ON THE PRODUCTION OF NITRIC OXIDE (NO) BY MACROPHAGES**

**Effect of leading compounds on the production of nitric oxide (NO) by healthy macrophages.**


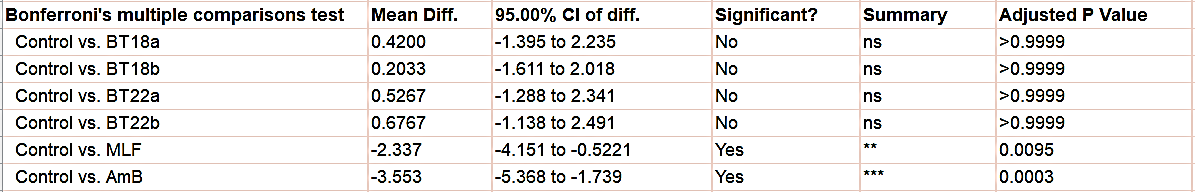


*Test was performed using GraphPad Prism version 8.0.2/2019 for Windows.*

**Effect of leading compounds on the production of nitric oxide (NO) by infected macrophages with reference *Leishmania amazonensis* LTB0016.**


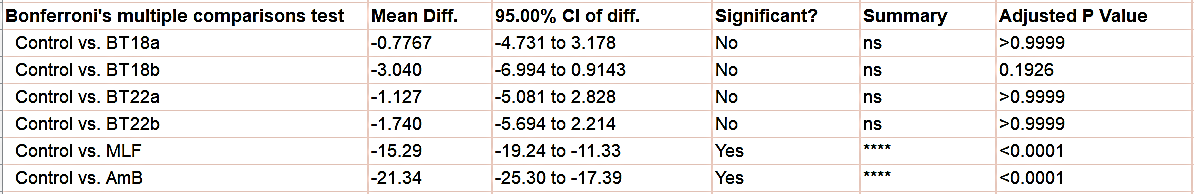


*Test was performed using GraphPad Prism version 8.0.2/2019 for Windows.*

**Effect of leading compounds on the production of nitric oxide (NO) by infected macrophages with *Leishmania amazonensis* VE98MR.**


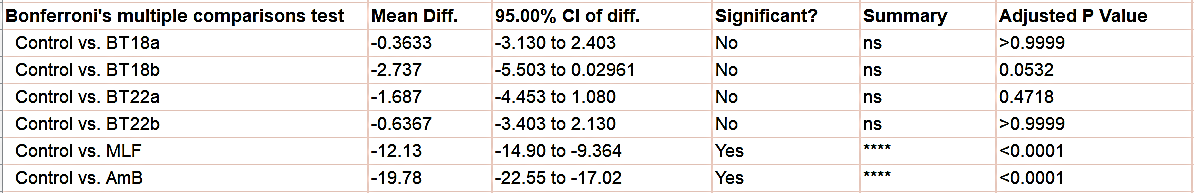


*Test was performed using GraphPad Prism version 8.0.2/2019 for Windows.*

**Effect of leading compounds on the production of nitric oxide (NO) by infected macrophages with *Leishmania amazonensis* VE2000MM.**


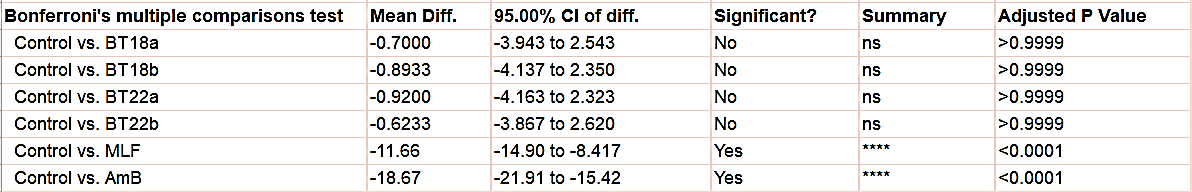


*Test was performed using GraphPad Prism version 8.0.2/2019 for Windows.*

**EFFECT OF LEADING COMPOUNDS ON THE PRODUCTION OF SUPEROXIDE ANION (O_2_^-^) BY MACROPHAGES**

**Effect of leading compounds on the production of superoxide anion (O_2_^-^) by healthy macrophages.**


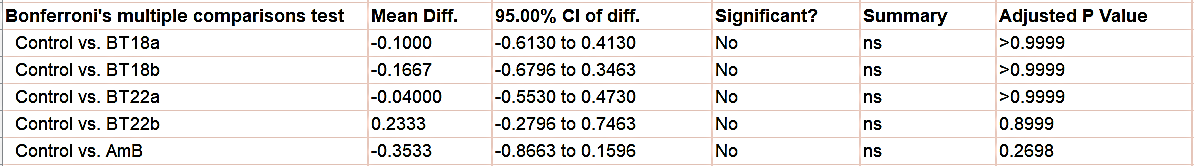


*Test was performed using GraphPad Prism version 8.0.2/2019 for Windows.*

**Effect of leading compounds on the production of superoxide anion (O_2_^-^) by infected macrophages with reference *Leishmania amazonensis* LTB0016.**


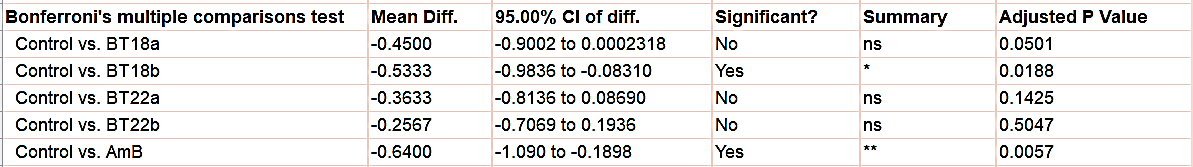


*Test was performed using GraphPad Prism version 8.0.2/2019 for Windows.*

**Effect of leading compounds on the production of superoxide anion (O_2_^-^) by infected macrophages with *Leishmania amazonensis* VE98MR.**


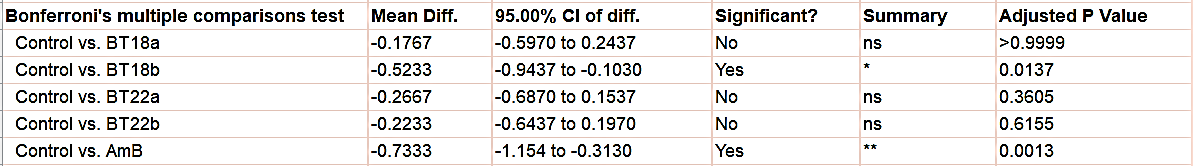


*Test was performed using GraphPad Prism version 8.0.2/2019 for Windows.*

**Effect of leading compounds on the production of superoxide anion (O_2_^-^) by infected macrophages with *Leishmania amazonensis* VE2000MM.**


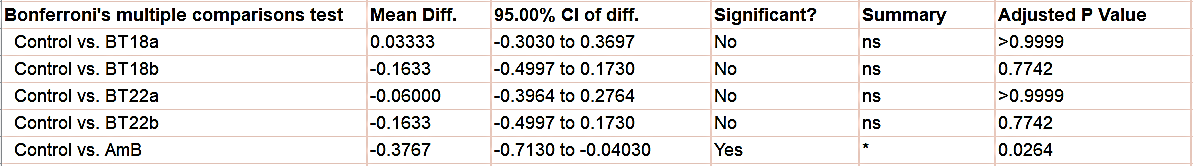


*Test was performed using GraphPad Prism version 8.0.2/2019 for Windows.*
