# Supplementary material for: Leishmanicidal Activity of Betulin Derivatives in Leishmania amazonensis; Effect on Plasma and Mitochondrial Membrane Potential, and Macrophage Nitric Oxide and Superoxide Production
Source: Microorganisms. 2021 Feb 4;9(2):320. doi: 10.3390/microorganisms9020320 (PMC7913927; doi:10.3390/microorganisms9020320)
Supplement: Supplementary file 1 [file microorganisms-09-00320-s001.zip › Alcazar et al. 2020_Supplementary Material I-III/Alcazar et al. 2020 Sup Mat III.pdf]

EFFECT OF LEADING COMPOUNDS ON PLASMA MEMBRANE POTENTIAL (ΔΨp)

Effect of leading compounds on plasma membrane potential (ΔΨp) in reference *Leishmania amazonensis* LTB0016 promastigotes.

Holm-Sidak's multiple comparisons test	Predicted (LS) mean diff.	Significant?	Summary	Adjusted P Value
Control vs. BT18a	-31.73	Yes	*	0.0414
Control vs. BT18b	-48.96	Yes	**	0.0034
Control vs. BT22a	-2.900	No	ns	0.7966
Control vs. BT22b	-12.51	No	ns	0.4761
Control vs. VAL	-32.59	Yes	*	0.0414
Control vs. CytB	-100.4	Yes	****	<0.0001
Control vs. DIDS	43.03	Yes	**	0.0080

Test was performed using GraphPad Prism version 8.0.2/2019 for Windows.

Effect of leading compounds on plasma membrane potential (ΔΨp) in *Leishmania amazonensis* VE98MR promastigotes isolated from patient.

Holm-Sidak's multiple comparisons test	Predicted (LS) mean diff.	Significant?	Summary	Adjusted P Value
Control vs. BT18a	-23.59	Yes	**	0.0059
Control vs. BT18b	-43.49	Yes	****	<0.0001
Control vs. BT22a	-2.749	No	ns	0.6649
Control vs. BT22b	-9.146	No	ns	0.2996
Control vs. VAL	-28.71	Yes	**	0.0016
Control vs. CytB	-61.83	Yes	****	<0.0001
Control vs. DIDS	33.37	Yes	***	0.0005

Test was performed using GraphPad Prism version 8.0.2/2019 for Windows.

Effect of leading compounds on plasma membrane potential (ΔΨp) in *Leishmania amazonensis* VE2000MM promastigotes isolated from patient.

Holm-Sidak's multiple comparisons test	Mean Diff.	Significant?	Summary	Adjusted P Value
Control vs. BT18a	-25.36	Yes	**	0.0024
Control vs. BT18b	-28.75	Yes	***	0.0009
Control vs. BT22a	-7.797	No	ns	0.1861
Control vs. BT22b	-12.37	No	ns	0.0871
Control vs. VAL	-15.17	No	ns	0.0504
Control vs. CytB	-51.10	Yes	****	<0.0001
Control vs. DIDS	24.82	Yes	**	0.0024

Test was performed using GraphPad Prism version 8.0.2/2019 for Windows.

EFFECT OF LEADING COMPOUNDS ON MITOCHONDRIAL MEMBRANE POTENTIAL (ΔΨm)

Effect of leading compounds on mitochondrial membrane potential (ΔΨm) in reference *Leishmania amazonensis* LTB0016 promastigotes.

Bonferroni's multiple comparisons test	Mean Diff.	95.00% CI of diff.	Significant?	Summary	Adjusted P Value
Control vs. BT18a	0.3467	0.03658 to 0.6568	Yes	*	0.0251
Control vs. BT18b	0.9167	0.6066 to 1.227	Yes	****	<0.0001
Control vs. BT22a	-0.09333	-0.4034 to 0.2168	No	ns	>0.9999
Control vs. BT22b	0.3367	0.02658 to 0.6468	Yes	*	0.0303
Control vs. FCCP	0.4633	0.1532 to 0.7734	Yes	**	0.0030
Control vs. OLIG	1.053	0.7432 to 1.363	Yes	****	<0.0001

Test was performed using GraphPad Prism version 8.0.2/2019 for Windows.

Effect of leading compounds on mitochondrial membrane potential (ΔΨm) in *Leishmania amazonensis* VE98MR promastigotes isolated from patient.

Bonferroni's multiple comparisons test	Mean Diff.	95.00% CI of diff.	Significant?	Summary	Adjusted P Value	A-?	
Control vs. BT18a	0.2467	-0.1781 to 0.6714	No	ns	0.5524	B	BT18a
Control vs. BT18b	0.7833	0.3586 to 1.208	Yes	***	0.0005	C	BT18b
Control vs. BT22a	-0.01333	-0.4381 to 0.4114	No	ns	>0.9999	D	BT22a
Control vs. BT22b	0.1367	-0.2881 to 0.5614	No	ns	>0.9999	E	BT22b
Control vs. FCCP	0.5800	0.1552 to 1.005	Yes	**	0.0061	F	FCCP
Control vs. OLIG	0.8033	0.3786 to 1.228	Yes	***	0.0004	G	OLIG

Test was performed using GraphPad Prism version 8.0.2/2019 for Windows.

Effect of leading compounds on mitochondrial membrane potential (ΔΨm) in *Leishmania amazonensis* VE2000MM promastigotes isolated from patient.

Bonferroni's multiple comparisons test	Mean Diff.	95.00% CI of diff.	Significant?	Summary	Adjusted P Value
Control vs. BT18a	0.1300	-0.2570 to 0.5170	No	ns	>0.9999
Control vs. BT18b	0.4100	0.02299 to 0.7970	Yes	*	0.0353
Control vs. BT22a	-0.04667	-0.4337 to 0.3403	No	ns	>0.9999
Control vs. BT22b	0.2000	-0.1870 to 0.5870	No	ns	0.7753
Control vs. FCCP	0.4267	0.03965 to 0.8137	Yes	*	0.0275
Control vs. OLIG	0.8267	0.4397 to 1.214	Yes	***	0.0001

Test was performed using GraphPad Prism version 8.0.2/2019 for Windows.

EFFECT OF LEADING COMPOUNDS ON THE PRODUCTION OF NITRIC OXIDE (NO) BY MACROPHAGES

Effect of leading compounds on the production of nitric oxide (NO) by healthy macrophages.

Bonferroni's multiple comparisons test	Mean Diff.	95.00% CI of diff.	Significant?	Summary	Adjusted P Value
Control vs. BT18a	0.4200	-1.395 to 2.235	No	ns	>0.9999
Control vs. BT18b	0.2033	-1.611 to 2.018	No	ns	>0.9999
Control vs. BT22a	0.5267	-1.288 to 2.341	No	ns	>0.9999
Control vs. BT22b	0.6767	-1.138 to 2.491	No	ns	>0.9999
Control vs. MLF	-2.337	-4.151 to -0.5221	Yes	**	0.0095
Control vs. AmB	-3.553	-5.368 to -1.739	Yes	***	0.0003

Test was performed using GraphPad Prism version 8.0.2/2019 for Windows.

Effect of leading compounds on the production of nitric oxide (NO) by infected macrophages with reference *Leishmania amazonensis* LTB0016.

Bonferroni's multiple comparisons test	Mean Diff.	95.00% CI of diff.	Significant?	Summary	Adjusted P Value
Control vs. BT18a	-0.7767	-4.731 to 3.178	No	ns	>0.9999
Control vs. BT18b	-3.040	-6.994 to 0.9143	No	ns	0.1926
Control vs. BT22a	-1.127	-5.081 to 2.828	No	ns	>0.9999
Control vs. BT22b	-1.740	-5.694 to 2.214	No	ns	>0.9999
Control vs. MLF	-15.29	-19.24 to -11.33	Yes	****	<0.0001
Control vs. AmB	-21.34	-25.30 to -17.39	Yes	****	<0.0001

Test was performed using GraphPad Prism version 8.0.2/2019 for Windows.

Effect of leading compounds on the production of nitric oxide (NO) by infected macrophages with *Leishmania amazonensis* VE98MR.

Bonferroni's multiple comparisons test	Mean Diff.	95.00% CI of diff.	Significant?	Summary	Adjusted P Value
Control vs. BT18a	-0.3633	-3.130 to 2.403	No	ns	>0.9999
Control vs. BT18b	-2.737	-5.503 to 0.02961	No	ns	0.0532
Control vs. BT22a	-1.687	-4.453 to 1.080	No	ns	0.4718
Control vs. BT22b	-0.6367	-3.403 to 2.130	No	ns	>0.9999
Control vs. MLF	-12.13	-14.90 to -9.364	Yes	****	<0.0001
Control vs. AmB	-19.78	-22.55 to -17.02	Yes	****	<0.0001

Test was performed using GraphPad Prism version 8.0.2/2019 for Windows.

Effect of leading compounds on the production of nitric oxide (NO) by infected macrophages with *Leishmania amazonensis* VE2000MM.

Bonferroni's multiple comparisons test	Mean Diff.	95.00% CI of diff.	Significant?	Summary	Adjusted P Value
Control vs. BT18a	-0.7000	-3.943 to 2.543	No	ns	>0.9999
Control vs. BT18b	-0.8933	-4.137 to 2.350	No	ns	>0.9999
Control vs. BT22a	-0.9200	-4.163 to 2.323	No	ns	>0.9999
Control vs. BT22b	-0.6233	-3.867 to 2.620	No	ns	>0.9999
Control vs. MLF	-11.66	-14.90 to -8.417	Yes	****	<0.0001
Control vs. AmB	-18.67	-21.91 to -15.42	Yes	****	<0.0001

Test was performed using GraphPad Prism version 8.0.2/2019 for Windows.

EFFECT OF LEADING COMPOUNDS ON THE PRODUCTION OF SUPEROXIDE ANION (O_2^-) BY MACROPHAGES

Effect of leading compounds on the production of superoxide anion (O_2^-) by healthy macrophages.

Bonferroni's multiple comparisons test	Mean Diff.	95.00% CI of diff.	Significant?	Summary	Adjusted P Value
Control vs. BT18a	-0.1000	-0.6130 to 0.4130	No	ns	>0.9999
Control vs. BT18b	-0.1667	-0.6796 to 0.3463	No	ns	>0.9999
Control vs. BT22a	-0.04000	-0.5530 to 0.4730	No	ns	>0.9999
Control vs. BT22b	0.2333	-0.2796 to 0.7463	No	ns	0.8999
Control vs. AmB	-0.3533	-0.8663 to 0.1596	No	ns	0.2698

Test was performed using GraphPad Prism version 8.0.2/2019 for Windows.

Effect of leading compounds on the production of superoxide anion (O_2^-) by infected macrophages with reference *Leishmania amazonensis* LTB0016.

Bonferroni's multiple comparisons test	Mean Diff.	95.00% CI of diff.	Significant?	Summary	Adjusted P Value
Control vs. BT18a	-0.4500	-0.9002 to 0.0002318	No	ns	0.0501
Control vs. BT18b	-0.5333	-0.9836 to -0.08310	Yes	*	0.0188
Control vs. BT22a	-0.3633	-0.8136 to 0.08690	No	ns	0.1425
Control vs. BT22b	-0.2567	-0.7069 to 0.1936	No	ns	0.5047
Control vs. AmB	-0.6400	-1.090 to -0.1898	Yes	**	0.0057

Test was performed using GraphPad Prism version 8.0.2/2019 for Windows.

Effect of leading compounds on the production of superoxide anion (O_2^-) by infected macrophages with *Leishmania amazonensis* VE98MR.

Bonferroni's multiple comparisons test	Mean Diff.	95.00% CI of diff.	Significant?	Summary	Adjusted P Value
Control vs. BT18a	-0.1767	-0.5970 to 0.2437	No	ns	>0.9999
Control vs. BT18b	-0.5233	-0.9437 to -0.1030	Yes	*	0.0137
Control vs. BT22a	-0.2667	-0.6870 to 0.1537	No	ns	0.3605
Control vs. BT22b	-0.2233	-0.6437 to 0.1970	No	ns	0.6155
Control vs. AmB	-0.7333	-1.154 to -0.3130	Yes	**	0.0013

Test was performed using GraphPad Prism version 8.0.2/2019 for Windows.

Effect of leading compounds on the production of superoxide anion (O_2^-) by infected macrophages with *Leishmania amazonensis* VE2000MM.

Bonferroni's multiple comparisons test	Mean Diff.	95.00% CI of diff.	Significant?	Summary	Adjusted P Value
Control vs. BT18a	0.03333	-0.3030 to 0.3697	No	ns	>0.9999
Control vs. BT18b	-0.1633	-0.4997 to 0.1730	No	ns	0.7742
Control vs. BT22a	-0.06000	-0.3964 to 0.2764	No	ns	>0.9999
Control vs. BT22b	-0.1633	-0.4997 to 0.1730	No	ns	0.7742
Control vs. AmB	-0.3767	-0.7130 to -0.04030	Yes	*	0.0264

Test was performed using GraphPad Prism version 8.0.2/2019 for Windows.
